# The Effectiveness of a Multi-Pronged Psycho-Social Intervention Among People With Mental Health and Epilepsy Problems - A Pre-Post Prospective Cohort Study Set in North India

**DOI:** 10.34172/ijhpm.2020.62

**Published:** 2020-05-12

**Authors:** Kaaren Mathias, Dale Corcoran, Pooja Pillai, Smita Deshpande, Miguel San Sebastian

**Affiliations:** ^1^Herbertpur Christian Hospital, Emmanuel Hospital Association, Uttarakhand, India.; ^2^Department of Epidemiology and Global Health, Umeå University, Umeå, Sweden.; ^3^Northland District Health Board, Whangarei, New Zealand.; ^4^Dr. RML Hospital and Post Graduate Institute of Medical Education & Research (PGIMER), New Delhi, India.

**Keywords:** Psychosocial, India, Mental Health, Intervention, Epilepsy

## Abstract

**Background:** In low- and middle-income settings, many people with mental health problems cannot or do not access psychiatric services. Few studies of people with epilepsy and mental problems have evaluated the effectiveness of a predominantly psycho-social intervention, delivered by lay community workers. The aim of this study was to assess the effectiveness of a community-based complex mental health intervention within informal urban communities while simultaneously addressing social determinants of mental health among disadvantaged people with severe and common mental disorders (CMDs), and epilepsy.

**Methods:** In this observational, prospective cohort study set in Uttarakhand, India, the lay-worker led intervention included psychoeducation, behavioural activation, facilitation of access to care, and facilitated psycho-social support groups. Participants were categorised as having a severe or CMD or epilepsy and assessed 5 times over 24 months using primary outcome measures, including the Patient Health Questionnaire (PHQ9) (severity of depression), the World Health Organization Disability Assessment Schedule (WHODAS 2.0), the Recovery Star, and scoring of a bespoke Engagement Index. Analysis included descriptive statistics as well as hierarchical linear regression models to report fixed effects as regression coefficients.

**Results:** Among the 297 (baseline) participants only 96 people (31%) regularly used psychotropic medication (at least 4 weeks) and over 60% could not or did not consult a psychiatrist at all in the study period. Nonetheless, people with CMDs showed a significant reduction in their depression severity (PHQ9: B=-6.94, 95% CI -7.37 to -6.51), while people with severe mental disorders (SMDs) showed a significant reduction in their disability score (WHODAS 2.0: B=-4.86, 95% CI - 7.14 to- 2.57). People with epilepsy also reduced their disability score (WHODAS 2.0: B=-5.22, 95% CI -7.29 to -3.15).

**Conclusion:** This study shows significant improvements in mental health, depression, recovery, disability and social engagement for people with common and SMDs, and epilepsy, through a community-based intervention that was nonpharmaceutical. It provides preliminary evidence of the value of predominantly psycho-social interventions implemented by lay health workers among people with limited or no access to psychiatric services.

## Background

Mental ill-health is the leading cause of years lived with disability^1^ and those affected are socially excluded, have reduced quality of life and a lower life expectancy.^2^ In India there is a life-time prevalence of 13.7% for mental ill-health and a treatment gap of 83%^3^ while the prevalence, and treatment gap for epilepsy is 0.6% and 70% respectively.^4^ Meanwhile, less than 1% of the national health budget is earmarked for mental health and epilepsy related services^5^ in a health system characterised by weak primary health services and a large, profit-focused and unregulated private sector.^6^ Particularly in rural areas there are very significant human resource gaps with one Government psychiatrist to cover several million population in many districts. Global responses addressing mental ill-health have primarily focused on provision of integrated psychiatric and drug treatments for those affected.^7^ While access to services is important, mental health and well-being require broader responses that also address social and development domains.^8^ Inclusion of community and social development approaches in global mental health programmes increases mental well-being, and access to care and improves outcomes.^7-9^ The use of lay workers has been a key approach to increase access to care in settings where there are few mental health professionals, and furthermore, to increase psycho-social care which is time consuming to offer.^7,10,11^

India’s National Mental Health Programme addresses epilepsy as well as mental ill-health^12^ and has had a primary focus on increasing access to care by increasing the numbers of psychiatrists and access to medicines.^13,14^ Other factors that influence access to care include non-Western explanatory frameworks, different idioms of distress and stigma. Recent community-based neuro-psychiatric interventions in India have demonstrated the value of the following programmatic components: training of lay workers to deliver care (task-sharing) to increase access to care in a setting where health professionals are scarce,^7,10,11^ improving knowledge and skills for self-care for those affected (psycho-education and community-based-rehabilitation), promoting social inclusion, and supporting access to care for mental, neurological and physical health problems.^15,16,17^ In most studies to date the contribution of lay workers has been in identification of people with mental or neurological problems and in facilitating access to care (task-sharing or shifting).^7,10,11^There are almost no studies in high or low income settings that evaluate the value of psychosocial support and care in settings where people with mental health problems cannot or do not access care.^18,19^

Globally there is an urgent call for impact evaluations of community-based mental health programmes in low resource and ‘real-life’ settings.^20^ The aim of this study was to assess the effectiveness of a multi-pronged community development and mental health intervention among people with mental health problems and epilepsy in Uttarakhand, North India.

## Methods

###  Setting

 This single-centred prospective cohort observational study with no control group was set in peri-urban communities in Uttarakhand, India and conducted between December 2015 and November 2017. When this study was implemented, the National Mental Health Programme had not been implemented in Uttarakhand state and 5 government psychiatrists provided services for the states’ 10 million people. The State Mental Health Institute in Selaqui (up to 2 hours travel from study locations) offered consultation and psychotropic medicines for minimal charge.

Burans, the implementing partner, is a community mental health partnership project. A baseline survey described 6% depression prevalence and those identified with depression described a treatment gap of 96%.^21^ The Burans field teams worked in the 3 sites of Sahaspur (rural), Mussoorie (small town) and Kanwali road (informal urban community).

###  Participants

 Participants were community members with epilepsy, mental health problems or psycho-social disability (PPSD) who were referred by community leaders and the government Accredited Social Health Activist workers or self-referred between September and November 2015. Participants were assessed for inclusion by trained community health workers during 2 home visits in December 2015. Training is described below (intervention section).

Inclusion criteria: People describing anxiety, unexplained somatic symptoms and/or depression who were able to fulfil most daily responsibilities were categorised as having a common mental disorder (CMD). People (or their caregivers) describing loss of social networks, lack of self-care and/or auditory hallucinations or delusions and unable to fulfil their daily responsibilities were assessed as having a severe mental disorder (SMD). People (or their caregivers) describing 3 or more discrete acute episodes of seizures in the past year were assessed as having epilepsy. Classifications were verified by either a trained psychiatric social worker or a health professional. Epilepsy was included in the study for 3 reasons: firstly, because in North India, the dominant explanatory framework for unexplained behaviour includes people with epilepsy along with other forms of mental illness; secondly, because the World Health Organization (WHO) groups neurological and psychiatric disorders together^5^ and thirdly, because the treatment and management of epilepsy is included in India’s National Mental Health programme and it is widely treated by psychiatrists.^22^

 Exclusion criteria: People who were aged under 14 years and people without a primary caregiver (as the intervention was strongly linked with caregivers).

###  Intervention

The intervention was built on the theoretical framework of community mental health competence with a focus on the 3 key domains of knowledge, safe social spaces and partnerships for action.^23^ The intervention sub-components were further developed through a literature review of existing community mental health and rehabilitation programmes from India and other low- and middle-income countries. A published realist case-study further details the theoretical basis of the intervention and the intervention process.^17^ Key mechanisms supporting the intervention include increasing mental health knowledge in dialogue, using peer-to- peer platforms and informal conversations, increasing safe social spaces (social inclusion) by building on informal and formal psycho-social support groups to increase critical reflexive conversations (conscientization) and supporting new relationships of peer support and friendship. Mechanisms supporting the third domain of partnerships for action involved engagement with rights based approaches to access entitlements and groups acting together for mental health.^17^

The intervention included individual and group components and was delivered by Burans community mental health workers (CMHW) who visited participants at their homes approximately fortnightly. CMHW were selected through a process of community consultation and were required to have completed high school. CMHW received 15 days of training using a validated training manual^24^ and a further 26 days of training during the implementation phase. Training topics included identifying and assessing people with mental ill-health, using assessment tools and group facilitation, psycho-social support, counselling skills, and use of a care plan.^25^Interventions were delivered by CMHW through 30–60 minutes home-based visits, every fortnightly where the same CMHW worked with the same participants. Intervention components had a core standard provision at an individual level, with additional bespoke components added by CMHW to respond to diverse socio-economic needs and participant engagement. Caregivers were a key part of the intervention and were present at nearly all CMHW– participant interactions.

 The Individual level components included:

- Psycho-education – increasing mental health knowledge in dialogue (not didactic instruction) with participants and carers, seeking to understand their explanatory frameworks.
- Active listening and motivational problem solving – supporting participants together with carers, to express emotions, identify and solve problems.
- Behavioural activation – reinforcing and supporting steps to recovery and increased engagement in daily responsibilities, together with caregivers.
- Promoting access to care: People with SMD and epilepsy and people with CMD scoring more than 15 on the PHQ9 (Patient Health Questionnaire) assessment as well as those not improved after 4 weeks of psycho-social support were supported to consult the nearest Government psychiatrist. CMHW would accompany PPSD to the doctor for 2 visits and also provided up to USD3 per person to assist with transport costs.
- Providing relevant and culturally appropriate support eg, accompanying an anxious PPSD to walk and drink ‘chai’ (tea) with a neighbour or accompanying them on public transport to the health provider.

The group level components comprised:

- Psycho-social support groups – these groups were offered to all participants and around 60% participated. They had mixed membership of PPSD and caregivers and comprised ten modules/meetings encouraging conscientization and critical reflection using a pictorial, story-based resource.^17^
- Financial inclusion opportunities – CMHW promoted household budgeting and savings and livelihood initiatives to psycho-social support groups. Four groups opted to form a micro-credit and saving Self Help Group. Over half of participants chose to join in a livelihood initiative (primarily sewing products for local sale).
- Accessing entitlements – education regarding Government schemes and use of India’s Right to Information Act was conducted with all participants.

Additional intervention components delivered to 40% of participants who were more socio-economically disadvantaged or who had a SMD included:

- Facilitating access to medical care – accompanied and provided financial support for transport or medicines to consult the Government psychiatrist for 2 visits (up to Rs200 = $US3).
- Greater visit frequency and duration.

CMHW documented intervention fidelity in the participant care plan after each visit with a summary of their discussion, intervention, and action plans for the ensuing fortnight. Attendance registers, and minutes of group meetings were also maintained.

 Four assessment tools were used to measure outcomes:

1. The “Recovery Star tool”^26^ is a subjective tool rating function from 1–10 (lowest to highest) in the following 9 areas: managing mental health, self-care, living skills, social networks, paid work, family relationships, addictive behaviour, household responsibilities, identity and self-esteem, trust and hope. Scoring was done collectively by participants (and/or carer), and CMHW using a locally developed Hindi-medium scoring rubric.
2. The “Engagement Index” was developed by participants, carers and the Burans team using outcome mapping approaches^27^ to reflect what mattered most to people affected in their daily functioning. It built on a series of 7 outcome statements as follows: the person participates in their own therapy; the person has good knowledge and understanding on mental health; the person contributes to household responsibilities, and to household income generation; the person has returned to employment/ school; the person participates in community leadership and the person speaks publicly about mental health. Participants and carers discussed and agreed together on the statements and scoring, with ‘Good’ (‘3’) ‘Medium’ (‘2’) and ‘Bad’ (‘1’). A composite score (equal weighting) was generated for each participant by summing their scores for the 7 statements (range 7–21).
3. The PHQ-9 is a nine-question probe on symptoms and severity of depression such as enjoyment of usual activities, and self-harm ideation which has been validated in India.^28,29^ Each symptom is scored as never (0), at times (1), often (2), all the time (3) and a high score indicates greater depressive symptoms.
4. The World Health Organization Disability Assessment Schedule (WHODAS 2.0) measures the severity of disability in activities of daily living, cognition, mobility, social ability and participation over the last 30 days using a 5-point Likert scale.^30^ It has strong properties in identifying PPSD and was co-developed in India and has been validated and used widely.^31^ We used the 12-item interviewer administered version.

###  Data Collection

 Burans team members were trained in consent taking and intervention-documentation over 3 days. Baseline data collected in December 2015 included socio-demographic details, carer arrangements and outcome assessments.

 All participants were assessed with the Recovery Star and the Engagement Index. Additionally, people with CMD were assessed with PHQ9 while those with SMD and epilepsy were assessed with the WHODAS 2.0 (T1) measured baseline, and (T2) was carried out 3 months later, while subsequent measures (T3-5) were carried out at 6-monthly intervals. While measures were completed by the CMHW, scoring and documentation was reviewed and validated by the team leader and monitoring officer, and care plan data were verified monthly by the monitoring officer who was independent of the daily implementation.

###  Data Analysis

 Data was collected on paper forms, translated into English and entered onto an electronic spreadsheet, anonymised and stored in password protected data files. Firstly, we descriptively analysed the participants’ socio-demographic profile. Secondly, given the panel structure of the data, hierarchical linear regression models were used for reporting the fixed effects as regression coefficients (β) with 95% confidence intervals. In these analyses, the health outcomes over time were regressed on time-varying covariates, with time representing the level 1 unit, which was nested within individuals (level 2 unit). First, an empty model was run without any predictors, then time was included, and in the final model, time and the individual-level predictors (gender, age, caste- an ancient Indian measure of social position, religion, occupation, house and caregiver) were incorporated.

A sub-analysis of the Engagement Index examined difference in mean scores for individual statements using a *t* test. Statistical significance of *P *<.05 was used for all measures.

## Results

**Figure 1:**
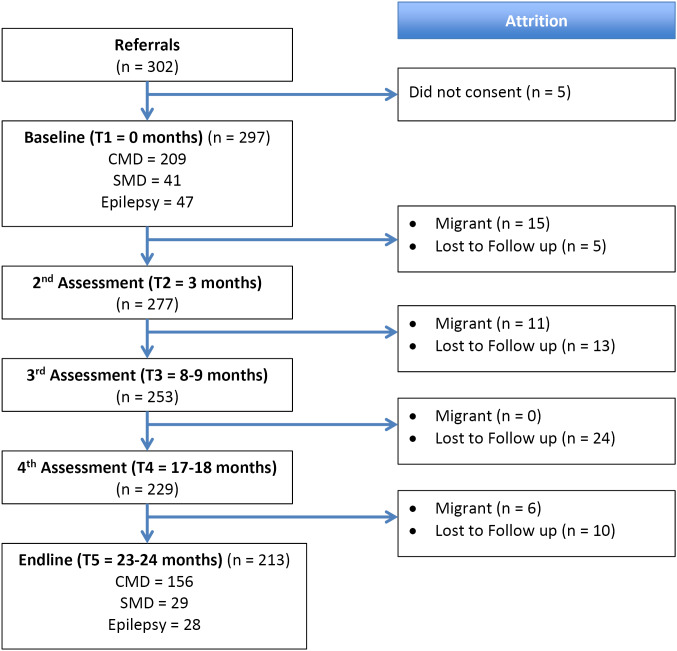
Overview of Study Sample and Attrition Over 24-Month Study Period.Abbreviations: CMD, common mental disorder; SMD, severe mental disorder.

Of the 302 people who were identified, 297 consented to participate (98.3%). Community leaders referred 110 participants to the team, while Accredited Social Health Activist workers referred 58 participants. The remaining 134 participants were self-referred. After 24 months of implementation, 213 people (71.7%) completed the end-line assessments. The median number of visits from a CMHW was 13.0 and the average number was 16.2. The sequence of the attrition of the 84 participants is summarised in Figure 1. Reasons for dropout included out-migration from the district (37%) and lost to follow-up (63%).

A profile of the participants assessed at baseline showed a higher representation of women, people over 60 years of age and socio-economically disadvantaged (unemployed and unskilled labourer) participants than the Dehradun district population^32^ (Table 1).

**Table 1:** Socio-Demographic Profile of Participants Recruited at Baseline

**Descriptive Variable**	**Male ** **No. (%)**	**Female ** **No. (%)**	**Total ** **No. (%)**
Total	117 (39.4)	180 (60.6)	297 (100)
Age (y)			
14-17	7 (6.0)	16 (8.9)	23 (7.7)
18-39	59 (50.4)	86 (47.8)	145 (48.8)
40-59	38 (32.5)	59 (32.7)	97 (32.7)
60+	13 (11.1)	19 (10.6)	32 (10.8)
Housing			
Temporary materials	40 (34.2)	73 (40.6)	113 (38.0)
Permanent materials	77 (65.8)	107 (59.4)	184 (62.0)
Occupation			
Professional	6 (5.1)	2 (1.1)	8 (2.7)
Skilled labour	11 (9.4)	12 (6.7)	23 (7.7)
Unskilled labour	46 (39.3)	42 (23.3)	88 (29.6)
Unemployed	42 (35.9)	49 (27.2)	91 (30.6)
Student/other	12 (10.3)	15 (8.3)	27 (9.1)
Homemaker	0 (0)	60 (33.3)	60 (20.2)
Main caregiver			
Parent	43 (36.8)	38 (21.1)	81 (27.3)
Spouse	48 (41.0)	99 (55.0)	147 (49.5)
Child	7 (6.0)	24 (13.3)	31 (10.4)
Other/other family	19 (16.3)	19 (10.6)	38 (12.7)
Religion			
Hindu	67 (57.3)	106 (58.9)	173 (58.2)
Muslim	50 (42.7)	73 (40.6)	123 (41.4)
Christian	0	1 (0.6)	1 (0.3)
Caste^a^			
General	40 (34.2)	52 (28.9)	92 (31.0)
OBC	57 (48.7)	83 (46.1)	140 (47.1)
SC/ST	20 (17.1)	45 (25.0)	65 (21.9)

Abbreviations: OBC, other backward castes; SC/ST, scheduled castes/tribes. ^a^ The 3 main caste classifications in the Indian Census: General referring to people from advantaged castes; OBC people from moderately oppressed castes and SC/ST. SC/ST refers to people from the most oppressed castes or of indigenous tribal ethnicity. Both SC and ST groups are considered systematically disadvantaged.

 The majority of participants were aged 18–49 years and more than two-thirds identified as being from the disadvantaged groups of ‘Other backward castes’ (OBC) and Scheduled castes/tribes (SC/ST). The relative disadvantage of this study’s participants was also evidenced by the fact that only 10.4% of participants were employed as professionals or in skilled labour and that 38% of participants were living in housing made of temporary materials.

As expected, baseline levels of disability and mental illness were greatest for people with SMD and least for people with CMD (Table 2). The difference in scores between end-line and baseline showed a statistically significant improvement in every measure of mental health, disability and recovery for participants with CMD, SMD and epilepsy. The effect sizes were large, particularly for people with CMD.

**Table 2:** Change in Means (SD) of Outcomes and Effect Sizes (β and 95% CI) From Baseline (T1) to End-line (T5) Adjusted for Covariates for CMD, SMD and Epilepsy

**People With CMD**s	**Baseline, n = 209**	**End-line, n = 156**	**Effect Size (95% CI)** ^c^
Recovery Star^a^	62.48 (15.37)	94.79 (7.25)	32.11 (30.28, 33.93)
PHQ9^b^	13.02 (2.90)	5.88 (2.70)	-6.94 (-7.37, -6.51)
Engagement Index mean^a^	11.16 (3.39)	18.47 (2.84)	7.17 (6.66, 7.68)
**People With SMDs **	**n = 41**	**n = 29**	
Recovery Star	36.98 (13.91)	63.23 (23.17)	23.90 (19.33, 28.47)
WHODAS 2.0^b^	35.15 (11.45)	27.17 (11.63)	-4.86 (-7.14, -2.57)
Engagement Index (mean^a^)	8.17 (2.30)	12.43 (4.14)	4.11 (2.95, 5.27)
**People With Epilepsy **	**n = 47**	**n = 28**	
Recovery Star^a^	58.32 (20.56)	86.52 (16.09)	26.30 (21.96, 30.64)
WHODAS 2.0^b^	23.83 (9.36)	16.71 (6.52)	-5.22 (-7.29, -3.15)
Engagement index mean^a^	10.08 (3.91)	14.32 (4.58)	3.00 (1.80, 4.19)

Abbreviations: WHODAS 2.0, World Health Organization Disability Assessment Schedule; CMDs, common mental disorders; SMDs, severe mental disorders; PHQ9, Patient Health Questionnaire. ^a^ Recovery Star and Engagement index: high score indicates greater level of function. ^b^ PHQ9 and WHODAS: high score indicates higher level of depression/disability. ^c^ Hierarchical multivariable linear regression adjusted for sex, age, caste, religion, occupation, house and caregiver.

 Of 297 people, only 123 (41.4%) visited a psychiatrist or neurologist at least once during the 24-month study period. Psychotropic medication had been utilised at least once by just 40.4% (n = 120) and 32.3% (n = 96) had used medication regularly (for a minimum of 4 weeks consecutively), meaning the majority of participants were primarily participating in psychosocial interventions.

Table 2 shows the change in means of the different outcomes and effect sizes from baseline (T1) to end-line (T5) adjusted for covariates for CMD, SMD, and epilepsy. In all 3 conditions and with all outcome measures, there was a statistically significant change between the 2 periods with increases in the Recovery star and the Engagement index and decreases in PHQ9 and WHODAS 2.0.

**Figure 2:**
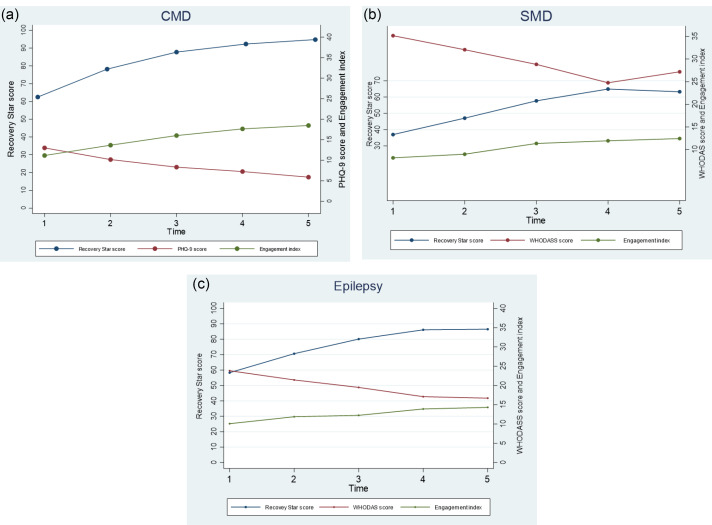
Trends in Mean Scores in the Different Outcomes (Recovery Star, PHQ9, Outcome Statements, WHODAS 2.0) According to the 3 Categories of Mental Health Problems: (a) CMD; (b) SMD and (c) Epilepsy. Abbreviations: WHODAS 2.0, World Health Organization Disability Assessment Schedule; CMDs, common mental disorders; SMDs, severe mental disorders; PHQ9, Patient Health Questionnaire.

The changes in scores measures across the 5 assessments are illustrated in Figure 2a for CMD, in Figure 2b for SMD and in Figure 2c for epilepsy. The trends showed improvements in scores between baseline and end-line with the largest improvements occurring in the first 12-18 months of participation.

People with CMD show a large reduction in their depression scores over time (PHQ9) and consistent improvement in their recovery and engagement scores. Figure 2b also demonstrates a steady increase in recovery measures, and a reduction in levels of disability measured by WHODAS 2.0 for people with SMD and epilepsy, however a plateau was observed between T4 and T5.

 Analysis of individual statements of the Engagement Index score (detailed in Methods) showed significant improvement in mean scores for every Engagement statement for people with CMD and in community participation and leadership, household contribution and social inclusion for people with SMD and epilepsy.

People with CMD, SMD, and epilepsy all showed statistically significant improvements (*P* < .05) in all outcome measures over the 5 time points.

## Discussion

 This 2-year prospective study demonstrates statistically significant improvements in mental health, recovery, disability and social engagement among people affected by common and SMDs or epilepsy, with a multi-pronged community-based mental health intervention. These outcomes were objectively and subjectively measured using validated psycho-metric tools with further verification through subjective measures scored by participants and carers together.

###  Contribution of Lay Workers

This paper underlines the value of lay worker psycho-social support among people with mental health problems and epilepsy where the majority of participants were not accessing psychiatric care. While there is good evidence supporting the contribution of lay workers in collaborative care for CMD in low- and middle-income country, an editorial reviewing next research steps to meet the needs for people with SMD in these countries underlined that there are almost no evaluations of community-based interventions among disadvantaged people with severe mental illness who have no access to care.^33^ This study addresses aspects of this gap with statistically significant evidence showing the benefit of psycho-social care even for those without psychiatric care.

CMHW in this study provided effective psycho-social care to people who would not otherwise access any care, underlining the contribution of non-specialist community workers (CMHW) in detection, psychosocial therapy, support and group interventions, which is evidenced in by a studies in high income countries showing that psycho-social interventions via non-specialist workers can significantly improve quality of life and mental health for people with severe mental illness.^19^ The role of CMHW in identifying and referring affected persons ie, ‘task shifting’ in the absence of professionals is a common characteristic of global mental health interventions.^34^ However, in this study, CMHW additionally performed roles not typically provided by a mental health professional such as accompanying to health services, home-visits and providing contextually relevant practical support that contributed to positive outcomes even for people with SMD or epilepsy.^35^ The CMHW contributions were broader and meshed with community ecology, development and dialogue^36^ and were different in substance to task-sharing as the ‘stop-gap’ measure framed in vertical global health interventions.^18^ Ongoing research and programmes that build on task-sharing in global mental health must expand understandings of the contributions of CMHW and evaluate these beyond the traditional narrow boundaries outlined for stepped-care and task-shifting.^18^

###  Value of Multi-pronged Intervention That Addressed Mental Health Determinants

This intervention responds to calls in global mental health for interventions that respond to social determinants for mental ill-health^8,25^ and did this by increasing social inclusion (safe social spaces) and by creating opportunities for participation in micro-credit/savings groups and livelihood initiatives (increased income, social, and financial inclusion). There is a strong link between social determinants of mental health such as poverty, income, social stigma and social connectedness and mental ill-health.^37^ Interventions in this study addressing broader social determinants of mental ill-health may have supported the positive outcomes through psychosocial support groups and peer to peer friendships. Furthermore, micro-credit and savings and livelihood opportunities may have improved economic status of participants. The relationship between socio-economic hardship and mental health is 2-way and mental health interventions can lead to improved economic outcomes for participants and accompanied by significant socio-economic improvement.^37^Additionally, intervention components to promote social participation and livelihood opportunities in this study may have also achieved positive results by increasing freedom of movement for women and challenging gender hierarchies,^38^as well as protecting participants from the negative impact of social drift and impoverishment which are prevalent among people with mental health problems in low-income contexts.^37^Proposed mediating mechanisms supporting the improved social inclusion and mental health include increased social contact, increased peer friendships and rehearsal of social and communication skills facilitated by participation in psycho-social support groups.^38^ Community mental health interventions must include and evaluate components addressing social determinants and equity, and include realist type components to understand what works for whom, under what circumstances.^7,20,39,40^

###  Building Knowledge Through Dialogue

A unique feature of this intervention was to increase knowledge and awareness around mental health and epilepsy through dialogue (instead of didactic instruction) with the participants and their carers. This platform of discussion allowed the production of knowledge in a relational process rather than the more traditional approach of psycho-education where knowledge flows unidirectionally from experts to community members.^41^Knowledge that is shared and debated can allow integration of unfamiliar medical knowledge with local explanatory frameworks and understandings.^42^ In this study, greater awareness about mental health, epilepsy and supported help-seeking may have facilitated the observed improvements in mental health status. In the context of North India where there are entrenched social hierarchies and the voice of the doctor/expert is elevated, democratising knowledge production seems particularly important to allow knowledge that can be actioned by individuals, and may have contributed to improvements in recovery and participation.^41^ Further programmes and research that examine how to build mental health literacy, engage positively with local knowledge and culture, and that seek to co-produce knowledge with people with lived experience in diverse Indian contexts is required.^43^

###  Methodological Considerations

 This study has some important limitations. As there was no control group, we cannot be sure the observed improvements can be attributed to the intervention only. We did not exclude people who were migrant labourers at enrolment and out-migration led to attrition of n = 31 participants reflecting the social disadvantage of participants. Overall more than one quarter of participants were lost to follow-up leading to a potential selection bias that could affect the interpretation of the estimates. However, a sub-analysis of enrolment data showed that the socio-demographic profile of participants who did not complete the study was not statistically different from those who completed the intervention. CMHW who delivered the intervention were not blinded to the outcome measures which could have increased information bias. As some scales were built on subjective responses by participants (Engagement Index and Recovery Star), scores may be inflated by social desirability bias. Further, the Recovery star measure was not developed for use by people with epilepsy and is not validated in this group. The most notable improvements in function occurred within the first 12–18 months of the intervention suggesting that a shorter intervention period could perhaps achieve similar outcomes but may still require ‘booster’ doses. Further research should measure outcomes in a controlled cohort study followed for several years after completion of the intervention to clarify whether improvements are sustained.

## Conclusion

 This study shows that a lay-health worker delivered multi-pronged mental health and community development intervention can achieve significant improvements in mental health, recovery and social participation for people with CMD, SMD and epilepsy in a poor resource setting. Further research should examine the role and contribution of lay workers in a more nuanced way, and further assess the effectiveness of psycho-social care in settings where there are few or no mental health professionals.

## Acknowledgements

 The intervention and data collection of this study was supported by members of the Burans team: Jeet Bahadur, Samson Rana, Kundan Goshan, Atul Goodwin, Pooja Bhatt, Kakul Krishna, Laxman Balan, Ronnie Issachar and the late Arun Sherring, as well as the hardworking community worker team are acknowledged here. Support to this work in logisitics and programme implementation from AKS-HOPE, OPEN, the Uttarakhand CHGN cluster and the Emmanuel Hospital Association is also acknowledged and appreciated.

## Ethical issues

 All participants provided written consent, with additional written consent provided by caregivers of all participants with SMD and those under 18 years of age. Identifiable data was only accessible by the relevant CMHW and 2 researchers, and data storage followed research ethics guidelines. Confidentiality was strictly maintained. Ethical approval for this study as protocol 181 was granted by the Institutional Ethics Committee of the Emmanuel Hospital Association, New Delhi in June 2015.

## Competing interests

 Authors declare that they have no competing interests.

## Authors’ contributions

 This study was conceived by KM who also wrote first and subsequent drafts, data acquisition and preliminary analysis was done by PP and DC, advanced analysis and supervision was completed by MSS, literature review and supervision by SD.

## Authors’ affiliations

^1^Herbertpur Christian Hospital, Emmanuel Hospital Association, Uttarakhand, India. ^2^Department of Epidemiology and Global Health, Umeå University, Umeå, Sweden. ^3^Northland District Health Board, Whangarei, New Zealand. ^4^Dr. RML Hospital and Post Graduate Institute of Medical Education & Research (PGIMER), New Delhi, India.

## Key Messages

**Implications for policy makers**

- Trained lay workers can provide effective psycho-social support for people with mental health problems in settings with limited access to psychiatric care.
- Psycho-social support that includes dialogue to increase knowledge on mental health, behavioural activation and participation in groups can improve mental health and social engagement.
- Psycho-social support can improve mental health and social participation, even for people with mental health problems who do not access regular psychiatric care.
- Disability associated with severe mental illness and epilepsy can be reduced with psychosocial support from lay workers.

**Implications for public**

In North India many people with mental health problems can access psycho-social support more easily than consult with the limited number of available state psychiatrists. Few studies from community settings evaluate the value of primarily psycho-social interventions among people with limited access to care. This study aimed to assess the effectiveness of a community-based ‘real-life’ intervention among disadvantaged people with severe and common mental disorders (CMD), and epilepsy. The intervention included home-based visits, building knowledge, encouraging daily activities and responsibilities (behavioural activation) and assisting people to visit the nearest Government psychiatrist if required. The 297 participants were assessed 5 times over 24 months using measures of mental health and disability. Participants reported improved mental health, increased community participation and reduced disability even though two-thirds of them never visited a mental health professional. This study supports the value of psycho-social interventions implemented by lay health workers for social improvement, even when people do not access psychiatric services.
